# Light-Stimulated IGZO Transistors with Tunable Synaptic Plasticity Based on Casein Electrolyte Electric Double Layer for Neuromorphic Systems

**DOI:** 10.3390/biomimetics8070532

**Published:** 2023-11-09

**Authors:** Hwi-Su Kim, Hamin Park, Won-Ju Cho

**Affiliations:** 1 Department of Electronic Materials Engineering, Kwangwoon University, Gwangun-ro 20, Nowon-gu, Seoul 01897, Republic of Korea; hwisu0811@naver.com; 2 Department of Electronic Engineering, Kwangwoon University, Gwangun-ro 20, Nowon-gu, Seoul 01897, Republic of Korea; parkhamin@kw.ac.kr

**Keywords:** optoelectronic synapses, synaptic device, indium–gallium–zinc–oxide thin film transistors, electric double layers, persistent photoconductivity

## Abstract

In this study, optoelectronic synaptic transistors based on indium–gallium–zinc oxide (IGZO) with a casein electrolyte-based electric double layer (EDL) were examined. The casein electrolyte played a crucial role in modulating synaptic plasticity through an internal proton-induced EDL effect. Thus, important synaptic behaviors, such as excitatory post-synaptic current, paired-pulse facilitation, and spike rate-dependent and spike number-dependent plasticity, were successfully implemented by utilizing the persistent photoconductivity effect of the IGZO channel stimulated by light. The synergy between the light stimulation and the EDL effect allowed the effective modulation of synaptic plasticity, enabling the control of memory levels, including the conversion of short-term memory to long-term memory. Furthermore, a Modified National Institute of Standards and Technology digit recognition simulation was performed using a three-layer artificial neural network model, achieving a high recognition rate of 90.5%. These results demonstrated a high application potential of the proposed optoelectronic synaptic transistors in neuromorphic visual systems.

## 1. Introduction

Recent advances in artificial intelligence (AI) technology have revolutionized various aspects of our daily lives, such as visual and language recognition as well as automatic driving [1,2,3]. However, the traditional von Neumann architecture, which separates the memory and computation modules, faces challenges in handling the increasing complexity of data, leading to issues of high energy consumption [4,5,6]. As an alternative, the concept of neuromorphic computing has emerged, aiming to mimic the human brain’s computing capabilities [7,8,9,10]. The human brain, with its approximately 10^15^ synapses interconnecting neurons, can perform complex functions, including thinking, perception, learning, memorizing, and decision making, with a remarkably low power consumption of approximately 20 W [11]. Emulating synapses is an essential requirement for realizing neuromorphic systems. Various types of synaptic devices, such as field-effect transistors [12,13], memristors [14,15], and phase-change memory [16,17], have been extensively investigated to emulate biological synaptic functions. However, many of these devices operate based on electrical signals, which have various limitations related to the processing speed, cross-talk problems, and bandwidth–connection density trade-offs [18,19]. In this regard, optically stimulated synaptic devices offer distinct advantages, including non-contact signal transmission, low cross-talk, low power consumption, and high bandwidth [19,20]. Furthermore, these devices can convert optical inputs into electrical outputs, enabling them to imitate the unique and important visual system of humans. The retinal, a light-sensitive layer that converts light signals into electrical spikes, plays a significant role in the human visual system, providing over 80% of the external information processed by the brain [21,22,23]. Until now, amorphous oxide semiconductors (AOSs) have received significant attention due to their distinctive persistent photoconductivity (PPC) effects, which are highly desirable for optoelectronic synaptic devices [20,21,24,25,26,27]. When an optical input is applied to the AOS channel, photo-generated electrons are generated through the ionization of oxygen vacancies within the subgap region, resulting in increased channel conductance. After the optical input is eliminated, the photo-generated electrons never rapidly dissipate through the process of recombination [28,29,30]. Importantly, the PPC effects of AOSs allow the conductance to be maintained over time, enabling the realization of biological plasticity, such as long-term potentiation, in optoelectronic synaptic devices [31,32]. Additionally, the recombination process, which is an intrinsic relaxation characteristic of PPC effects, can be controlled to effectively regulate synaptic plasticity. For example, Sun et al. demonstrated the transformation of plasticity from short-term memory (STM) to long-term memory (LTM) using an electric double-layer (EDL) effect in sodium alginate-based zinc oxide nanowire synaptic transistors [33].

In this study, we propose an optoelectronic synaptic transistor based on indium–gallium–zinc oxide (IGZO) with a casein electrolyte-based electric double layer (EDL) as the gate insulator. The casein–EDL plays a key role as a modulator of synaptic plasticity based on the EDL effect. We have successfully implemented essential synaptic functions, including an excitatory post-synaptic current (EPSC), paired-pulse facilitation (PPF), spike rate-dependent plasticity (SRDP), and spike number-dependent plasticity (SNDP), by utilizing light spikes as pre-synaptic stimuli. Furthermore, we evaluated the transition from STM to LTM by modulating synaptic plasticity with the synergy of light spikes and the EDL effect. Finally, we constructed a three-layer artificial neural network (ANN) model and performed recognition simulations using the Modified National Institute of Standards and Technology (MNIST) handwritten digit dataset. The obtained results demonstrate a high utilization potential of the proposed optoelectronic synaptic transistors as building blocks for neuromorphic visual systems.

## 2. Materials and Methods

### 2.1. Casein Electrolyte Solution Preparation

The casein electrolyte solution was prepared via the microwave irradiation (MWI) process, which is more effective in energy transfer to the target. In the first step, the casein powder (technical grade, Sigma–Aldrich, Seoul, Republic Korea) of 3 wt.% was added to the deionized water diluted with acetic acid (purity > 99%, Sigma–Aldrich) of 3 wt.%. The obtained mixture was completely synthesized into a casein electrolyte solution using the MWI process. The MWI process was carried out for 5 min at a microwave frequency of 2.45 GHz with a power of 250 W. In the final step, to remove impurities, the solution was filtered using a 5 μm pore-sized polytetrafluoroethylene syringe filter (Whatman International Ltd., Maidstone, UK).

### 2.2. Device Fabrication

To fabricate the IGZO optoelectronic synaptic transistors with casein electrolyte-based EDL, transparent and flexible polyethylene naphthalate (PEN) substrates were affixed to carrier glass substrates using a cool-off-type adhesive before starting the processes. An indium–tin–oxide (ITO; In_2_O_3_:SnO_2_ = 9:1 mol.%, THIFINE Co., Ltd., Incheon, Republic of Korea) film with 300 nm thickness was deposited onto the PEN substrate using radio frequency (RF) magnetron sputtering as the gate electrode. The ITO sputtering conditions were a chamber pressure of 3 mTorr, a flow rate of 20 sccm for Ar, and an RF power of 100 W. To form the EDL as a gate insulator, the prepared casein electrolyte solution was spin-coated onto the ITO/PEN substrates at 3000 r/min for 30 s. Subsequently, the coated film was air-dried at 25 °C for 24 h. To form the channel layer, an IGZO (In_2_O_3_:Ga_2_O_3_:ZnO = 4:2:4.1 mol.%, THIFINE Co., Ltd.) film with 50 nm thickness was deposited through a shadow mask using RF magnetron sputtering. The IGZO sputtering conditions were a chamber pressure of 6 mTorr, a flow rate of 30 sccm for Ar, and RF power of 100 W. The channel dimensions were 1000 μm × 80 μm. With RF magnetron sputtering, a 150 nm thick ITO film deposition was followed through the shadow mask to form the source/drain electrodes with dimensions 1000 μm × 200 μm. Finally, the fabricated transistors onto the PEN substrate were easily separated from the carrier glass at low temperatures (<5 °C).

### 2.3. Devices Characterization

The electrical characteristics and synaptic properties of the proposed devices were measured using the Agilent 4156B precision semiconductor parameter analyzer (Hewlett–Packard Corp., Palo Alto, CA, USA). For applying various synaptic spike stimuli, the Agilent 8110A pulse generator (Hewlett–Packard Corp., USA) was utilized and a UV LED with a wavelength of 395 nm was used as the light signal input. The Fourier transform infrared (FTIR) spectroscopy (IFS66v/s and Hyperion3000, Bruker Optics, Billerica, MA, USA) was used to examine the chemical compositions in the casein electrolyte film. The capacitance–frequency (C–*f*) characteristic was characterized by the Agilent 4284A precision LCR meter (Hewlett–Packard Corp., USA). All measurements were conducted with the device placed in the metallic dark box to shield it from external effects, such as light and electrical noise.

## 3. Results and Discussion

**Figure 1:**
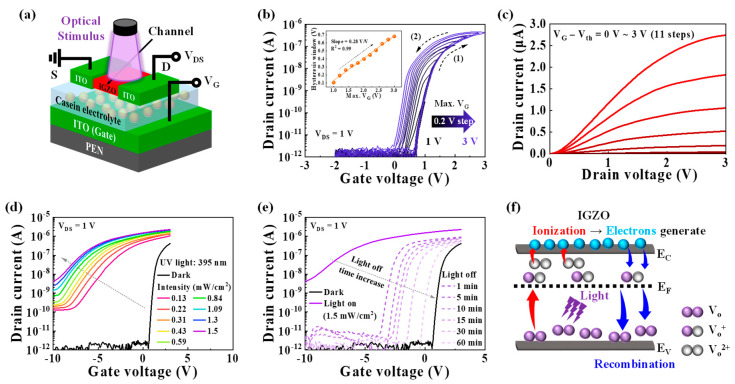
(**a**) Schematic image illustrating the structure of the IGZO optoelectronic synaptic transistor with a casein electrolyte−based EDL. (**b**) Double−sweep transfer curves of the optoelectronic synaptic transistor with increasing Max. V_G_ values from 1 to 3 V (in 0.2 V increments) at a constant V_DS_ of 1 V. Inset: extracted hysteresis window. (**c**) Output curves of the optoelectronic synaptic transistor. (**d**) Transfer curves of the optoelectronic synaptic transistor obtained under dark conditions and UV light illumination at different light intensities from 0.13 to 1.5 mW/cm^2^. The UV light wavelength is 395 nm. (**e**) Transfer curves that shift back to the initial (dark) curve over time after the light is turned off. (**f**) Mechanism of the oxygen vacancy ionization and recombination in the IGZO channel.

Figure 1a illustrates the schematic diagram of the IGZO optoelectronic synaptic transistor with a casein electrolyte-based EDL. The optical image is shown in Figure S1a (Supplementary Materials). In this device, the pre-synapse corresponds to the light source, the post-synapse corresponds to the IGZO channel, and the synaptic cleft is represented by the casein electrolyte. The chemical composition of the casein electrolyte was evaluated using Fourier transform infrared (FT-IR) spectroscopy, as shown in Figure S2a (Supplementary Materials). The obtained FT-IR spectra confirmed the presence of abundant –OH groups within the casein electrolyte, contributing to high proton conductivity [12,26]. These groups facilitate the accumulation of protons at the casein electrolyte/IGZO interface under an external electric field, resulting in a significant EDL capacitance [26]. The C–*f* characteristics of the casein electrolyte were measured using an ITO/casein/ITO sandwich structure, as shown in Figure S2b (Supplementary Materials). The casein electrolyte exhibited a large capacitance of 2.67 μF/cm^2^ at 100 Hz, which decreases with increasing frequency. This behavior can be attributed to the accumulation of protons at the interfaces, forming the EDL at lower frequencies [34]. The double-sweep transfer curves of the IGZO optoelectronic synaptic transistor with the casein EDL are presented in Figure 1b, where the maximum gate voltage (Max. V_G_) was varied from 1 to 3 V at a fixed drain voltage (V_DS_) of 1 V. The transfer curves exhibit a counterclockwise hysteresis window that increases with increasing Max. V_G_. The hysteresis window increased from 0.1 to 0.68 V with a slope of 0.28 *v*/*v* (inset of Figure 1b). This hysteresis characteristic is attributed to the EDL effect of the casein electrolyte. During the V_G_ forward sweep, mobile protons with slow polarization inside the casein electrolyte accumulate at the casein electrolyte/IGZO interface. As the V_G_ backward sweep begins, the protons gradually diffuse back in the opposite direction. Consequently, increasing Max. V_G_ induces a greater accumulation of protons at the interface, resulting in the widening of the counterclockwise hysteresis window. The output curves depicted in Figure 1c demonstrate good ohmic contact and normal n-type transistor characteristics. Figure 1d shows the transfer curves under dark conditions and UV light (395 nm) illumination with increasing intensity from 0.13 to 1.5 mW/cm^2^ at a constant V_DS_ of 1 V. As the light intensity increases, the drain current significantly increases, and the threshold voltage shifts negatively. As the time elapses after the light is turned off, the transfer curve is gradually shifted back to the initial curve in the dark, as depicted in Figure 1e. Even after 60 min, the drain current remains higher compared to the initial curve. This phenomenon is attributed to the PPC effects of the IGZO channel. The PPC effects result from the ionization and recombination of oxygen vacancies (V_O_), as illustrated in Figure 1f. The V_O_ acts as a localized state in the deep subgap region close to the valence band maximum. Under optical stimulation, the V_O_ ionizes into two shallow donor states, namely single-ionized V_O_ (V_O_^+^) and double-ionized V_O_ (V_O_^2+^), leading to the generation of electrons and an increase in channel conductance. Even after the optical stimulation is eliminated, the photo-generated electrons gradually dissipate through recombination, which reduces the channel conductance [27,28,35]. In the IGZO optoelectronic synaptic transistor with a casein EDL, the synaptic behavior can be implemented through the PPC effects of the IGZO channel.

**Figure 2:**
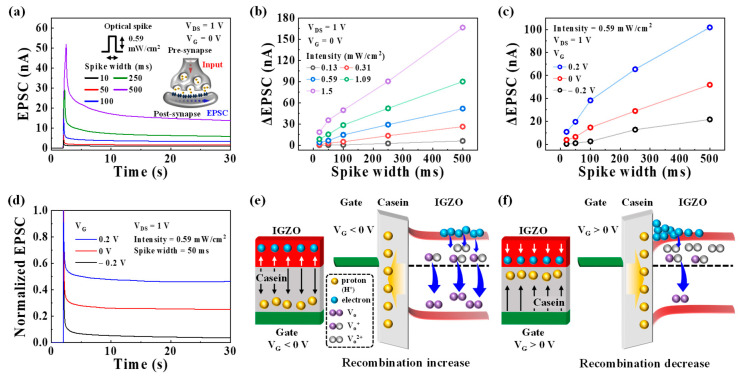
(**a**) EPSC triggered by a single pre-synaptic optical spike (395 nm, 0.59 mW/cm^2^) with various spike widths ranging from 20 to 500 ms at V_G_ of 0 V. Inset: illustration of a biological synapse. (**b**) ΔEPSC plotted against the light intensity ranging from 0.13 to 1.5 mW/cm^2^ at V_G_ of 0 V for different optical spike widths. (**c**) ΔEPSC plotted at different V_G_ values (−0.2 V, 0 V, and 0.2 V) with an optical spike (0.59 mW/cm^2^, 50 ms). (**d**) Normalized EPSC induced by the optical spike (0.59 mW/cm^2^, 50 ms) under different V_G_ values (−0.2 V, 0 V, and 0.2 V). Mechanisms of the recombination reactions that occur at (**e**) negative and (**f**) positive V_G_ values.

In the nervous system, synaptic plasticity serves as a critical function for complex information transmission, acquisition, and memory. These functions are facilitated by changes in synaptic weight (synaptic plasticity). In a biological synapse, the initiation of an action potential in the pre-synaptic neuron triggers the opening of Ca^2+^ channels, leading to the release of chemical neurotransmitters to the postsynaptic neuron. When these neurotransmitters bind to their receptors on the postsynaptic neuron, it results in the opening of ion channels and facilitates signal transmission [36], as illustrated in Figure S3a (Supplementary Materials). During this process, a transient current, known as an EPSC, is generated, leading to a change in the synaptic weight [34]. Figure 2a displays the typical EPSC induced by a single pre-synaptic optical spike (395 nm, 0.59 mW/cm^2^) with a variable spike width (ranging from 20 to 500 ms) at V_G_ of 0 V. EPSC was read with a constant V_DS_ of 1V. The EPSCs corresponding to different light intensities are shown in Figure S4 (Supplementary Materials). In the studied device, the EPSC is generated by the rapid increase in the number of photo-generated electrons when the IGZO channel is exposed to the optical spike. Subsequently, these photo-generated electrons gradually dissipate through recombination, resulting in the decay of the EPSC. This process is shown in Figure S3b (Supplementary Materials). Figure 2b presents the changes in the EPSC (ΔEPSC) obtained for different pre-synaptic optical spike widths and light intensities. ΔEPSC increases with higher light intensity and longer spike width because a greater number of electrons are generated within the IGZO channel in response to the stronger and longer light stimuli. This behavior exhibits a remarkable resemblance to the EPSC observed in biological synapses, indicating that the fabricated optoelectronic transistors can effectively mimic synaptic plasticity. Moreover, by integrating the EDL effect, synaptic plasticity can be modulated by controlling the recombination of photo-generated electrons in the IGZO optoelectronic synaptic transistor with a casein EDL. Figure 2c shows the ΔEPSC plots obtained at various V_G_ values (−0.2 V, 0 V, and 0.2 V) with a pre-synaptic spike (0.59 mW/cm^2^) for different spike widths. The corresponding EPSC is depicted in Figure S5 (Supplementary Materials). As the applied V_G_ increases under the same pre-synaptic optical spike conditions, the ΔEPSC magnitude increases. Figure 2d displays the normalized EPSC triggered by a synaptic optical spike (0.59 mW/cm^2^, 50 ms) at different V_G_ values. The residual EPSC level after the optical spike is higher at larger V_G_ values. These phenomena occur due to the influence of V_G_ on the migration of protons within the casein EDL, thereby affecting the recombination of photo-generated electrons. As shown in Figure 2e, at negative V_G_, protons move away from the casein/IGZO interface, leading to an increased recombination of photo-generated electrons. Consequently, the lower ΔEPSC and normalized residual EPSC are observed. Conversely, at positive V_G_ values, the EDL effect caused by the accumulation of protons at the casein/IGZO interface reduces recombination [37], as illustrated in Figure 2f. As a result, the higher ΔEPSC and normalized residual EPSC are obtained. Thus, the EDL effect with V_G_ serves as a synaptic plasticity modulator in the optoelectronic synaptic transistor.

**Figure 3:**
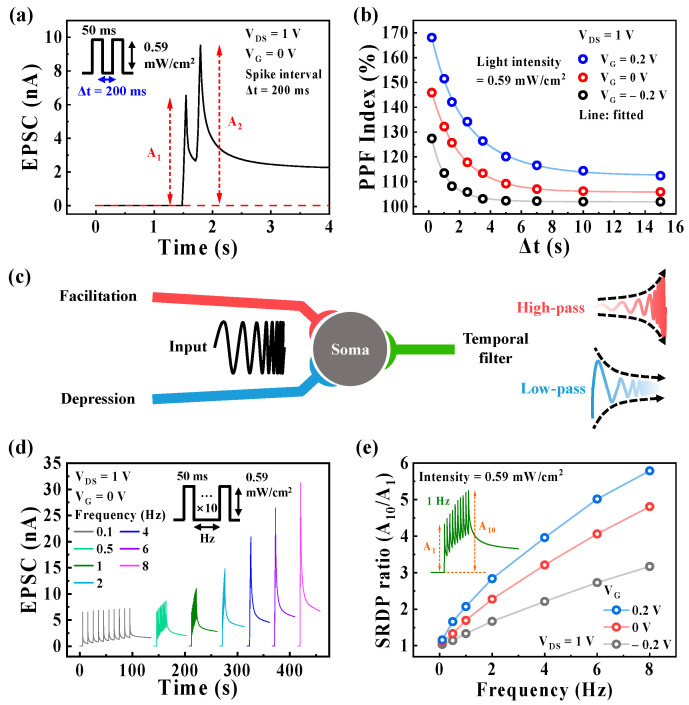
(**a**) EPSCs in response to the paired pre-synaptic optical spikes (0.59 mW/cm^2^, 50 ms) with a time interval (Δt) of 200 ms at V_G_ of 0 V. (**b**) PPF index (A_2_/A_1_ × 100) plotted as a function of Δt from 200 ms to 15 s at different V_G_ values (−0.2 V, 0 V, and 0.2 V). (**c**) Schematic of the temporal filter behaviors in the nervous system. (**d**) SRDP behavior triggered by the sequential 10 pre-synaptic optical spikes (each spike: 0.59 mW/cm^2^, 50 ms) with an increasing spike frequency from 0.1 to 8 Hz under V_G_ of 0 V. (**e**) SRDP ratio (A_10_/A_1_) plotted versus the pre-synaptic optical spike frequency at different V_G_ values (−0.2 V, 0 V, and 0.2 V). The inset denotes the SRDP behavior at 1 Hz under V_G_ of 0 V.

Synaptic plasticity can be classified into short-term plasticity (STP) and long-term plasticity (LTP) based on the retention time of the synaptic weight. The precise time boundary between STP and LTP is not defined; however, STP generally refers to the brief strengthening of synaptic weights lasting from milliseconds to seconds, whereas LTP involves the prolonged strengthening of synaptic weights lasting from seconds to years [22]. PPF is a typical indicator of STP and is crucial for processing temporal information, such as visual or auditory signals [38,39,40]. PPF is observed when the second EPSC (A_2_) increases compared to the first EPSC (A_1_) when the second spike closely follows the previous spike. The PPF index is defined as the ratio between the two triggered EPSCs (PPF index = A_2_/A_1_ × 100). Figure 3a shows the EPSC triggered by the paired pre-synaptic optical spikes (0.59 mW/cm^2^, 50 ms) at Δt of 200 ms with V_G_ of 0 V and was read with a constant V_DS_ of 1 V. The EPSCs triggered by the paired optical spikes at different V_G_ values (−0.2 V and 0.2 V) are presented in Figure S6 (Supplementary Materials). Two EPSCs were observed in response to the two successive pre-synaptic optical spikes, with A_2_ being greater than A_1_ owing to the accumulation of unrecombined photo-generated electrons from the first light spike in the IGZO layer, contributing to the A_2_ evoked by the second spike. Figure 3b displays the calculated PPF index for different Δt values ranging from 200 ms to 15 s under various V_G_ values (−0.2 V, 0 V, and 0.2 V). It shows that the PPF index increases with shorter Δt and decreases with longer Δt. Moreover, the PPF index increases at higher V_G_ values, indicating that synaptic plasticity facilitation can be modulated by controlling V_G_. For instance, at the shortest Δt of 200 ms, the PPF indexes obtained at V_G_ of −0.2 V, 0 V, and 0.2 V are 127.4%, 145.9%, and 168.1%, respectively. The calculated PPF indexes were fitted using Equation (1), which represents a double-exponential decay relationship [41]:

(1) $$PPF=C_{1}exp\left(\frac{-\Delta t}{\tau_{1}}\right)+C_{2}exp\left(\frac{-\Delta t}{\tau_{2}}\right)+A$$

where C_1_ and C_2_ are the initial acceleration magnitudes corresponding to the fast and slow phases, *A* is the constant, and τ_1_ and τ_2_ are the relaxation times of each phase. The estimated τ_1_ and τ_2_ values are 0.68 s and 1.58 s at a V_G_ of −0.2 V, 0.99 s and 2.04 s at a V_G_ of 0 V, and 1.04 s and 2.87 s at a V_G_ of 0.2 V.

Additionally, we estimated the energy consumption per spike event using the formula *I_peak_* × *t* × *V*, where *I_peak_*, *t*, and *V* are the peak EPSC value, optical spike width, and reading voltage (V_DS_), respectively [42,43]. The lowest energy consumption calculated for the EPSC is shown in Figure S7a (Supplementary Materials). The calculated energy consumption decreases as the pre-synaptic optical spike width decreases, with the lowest energy consumption being approximately 1.1 pJ (*I_peak_* = 56.4 pA, *t* = 20 ms, and *V* = 1 V), as shown in Figure S7b (Supplementary Materials). Table 1 shows the performance comparisons between the proposed IGZO optoelectronic synaptic transistor and various oxide-based optoelectronic synaptic devices.

Furthermore, in the human nervous system, synapses act as high-pass or low-pass filters for information transmission, depending on the STP with facilitation or depression [48], as illustrated in Figure 3c. Figure 3d displays the SRDP behavior with EPSCs triggered by 10 pre-synaptic optical spikes (0.59 mW/cm^2^, 50 ms) at different frequencies (ranging from 0.1 to 8 Hz) under V_G_ of 0 V and was read with a constant V_DS_ of 1 V. The SRDP behaviors for different V_G_ values (−0.2 V and 0.2 V) are shown in Figure S8 (Supplementary Materials). The EPSCs evoked by the sequential spikes increase with increasing spike frequency, indicating that the studied device functions as a high-pass filter, allowing the passage of high-frequency signals beyond a specific cutoff value. Figure 3e depicts the SRDP ratio versus frequency plotted for various V_G_ values. The SRDP ratio is obtained by dividing the final EPSC (A_10_) by the first EPSC (A_1_). The SRDP ratio increases with increasing both the spike frequency and V_G_. For example, at a spike frequency of 8 Hz, the SRDP ratios for a V_G_ of −0.2 V, 0 V, and 0.2 V are 3.16, 4.8, and 5.79, respectively. Thus, in our optoelectronic synaptic transistor, the cutoff value of the high-pass filter can be controlled by modulating synaptic plasticity through the EDL effect with V_G_.

**Figure 4:**
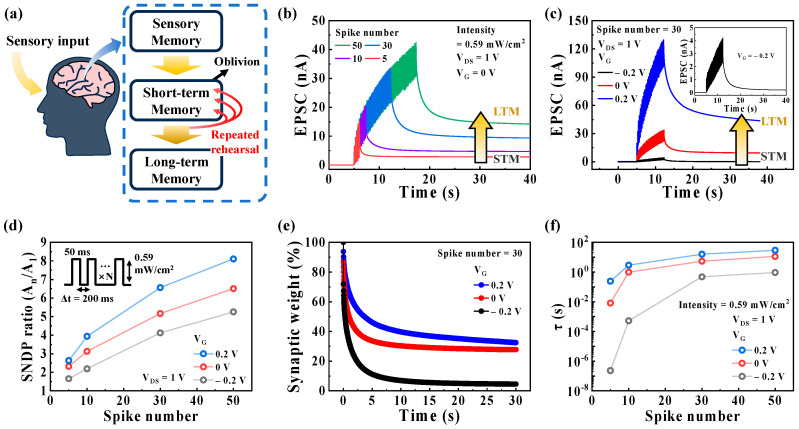
(**a**) Schematic illustration of the memory process of transitioning from STM to LTM in the human brain. (**b**) SNDP behaviors triggered by a varying number of pre-synaptic optical spikes (395 nm, 0.59 mW/cm^2^) with a Δt of 200 ms at V_G_ of 0 V. (**c**) SNDP characteristics with 30 spikes (0.59 mW/cm^2^, 50 ms) under different V_G_ values (−0.2 V, 0 V, and 0.2 V). The transition from STM to LTM is achieved by increasing the number of spikes and V_G_. (**d**) SNDP ratio (A_n_/A_1_) plotted as a function of the spike number at different V_G_ values (−0.2 V, 0 V, and 0.2 V). (**e**) Decay curves of the synaptic weight, referred to as the normalized channel conductance after applying the final spike, under different V_G_ values (−0.2 V, 0 V, and 0.2 V). (**f**) The obtained retention time (τ) plotted as a function of the number of spikes and V_G_.

In addition, STP can be converted to LTP, representing LTM in terms of synaptic memory levels, through repeated rehearsal events, which are believed to be essential for learning and memory in the human brain [22,39,42,43]. Figure 4a illustrates the memory process in the human brain, as proposed by Atkinson and Shiffrin. Figure 4b shows the EPSCs triggered by the different numbers of repeated optical spikes (395 nm, 0.59 mW/cm^2^) with Δt of 200 ms at V_G_ of 0 V and was read with a constant V_DS_ of 1 V, which is called SNDP behavior, indicating the emulation of the STM to LTM transition. The SNDP behaviors under different V_G_ values are shown in Figure S9 (Supplementary Materials). The EPSC increases with a higher number of spikes. Furthermore, the STM to LTM transition is implemented by controlling V_G_ from −0.2 to 0.2 V, as shown in Figure 4c. The SNDP ratio, calculated by the ratio of A_n_/A_1_, is plotted against V_G_ and the number of optical spikes in Figure 4d. The SNDP ratio improves with an increase in the number of optical spikes and V_G_. For instance, when the number of spikes increases from 5 to 50, the SNDP ratio increases from 1.65 to 5.25 at a V_G_ of −0.2 V, from 2.32 to 6.5 at a V_G_ of 0 V, and from 2.63 to 8.11 at a V_G_ of 0.2 V.

Figure 4e shows the decay curves of the synaptic weight obtained at different V_G_ values. A decay of the synaptic weight is described as the normalized channel conductance following the final spike. The synaptic weight characteristics of our synaptic transistor exhibit a rapid initial decay followed by a slower decay, which is similar to that of human memory. As shown in Figure S10 (Supplementary Materials), the decay of the synaptic weight can be fitted with a stretched exponential function [49]:

(2) $$\frac{\Delta G(t)}{\Delta G_{0}}=exp\left[-{\left(\frac{t}{\tau }\right)}^{\beta }\right]$$

where Δ*G*(*t*) = *G*(*t*) − *G*_i_, and Δ*G*_0_ = *G*_0_ − *G*_i_, *G*(*t*), *G*_0_, and *G*_i_ are the time-dependent, maximum, and initial (before the optical spike) conductance, respectively, and τ and *β* represent the characteristic relaxation time and the stretch index varying between 0 and 1, respectively. This equation is analogous to the Ebbinghaus forgetting curve, which postulates the diminishing retention of human memory over time [7,50]. As the retention time increases, the memory level improves, resulting in the conversion from STM to LTM [51]. Figure 4f shows the retention times (τ) obtained from the fitting curves of the optoelectronic synaptic transistor, where τ increases with the number of spikes and V_G_. In 50 optical spikes, the τ values reaches 0.93 s, 11.2 s, and 29.2 s at V_G_ of −0.2 V, 0 V, and 0.2 V, respectively. The τ value at V_G_ of 0.2 V is 31.4 times higher than that obtained at −0.2 V. These characteristics indicate that the memory level can be enhanced through repeated optical stimulation in our synaptic transistor. Additionally, the STM to LTM transition can be achieved by modulating the memory level through adjusting V_G_ related to the EDL effect.

**Figure 5:**
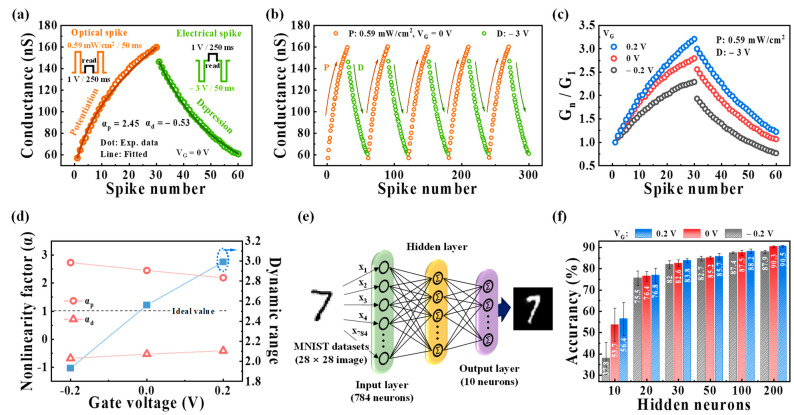
(**a**) Synaptic weight update characteristics applied by the optical potentiation spikes (0.59 mW/cm^2^, 50 ms) and electrical depression spikes (−3 V, 50 ms). The inset illustrates the applied potentiation and depression stimuli conditions. (**b**) Endurance characteristics observed during five cycles. (**c**) Normalized synaptic weights obtained at different V_G_ (−0.2 V, 0 V, and 0.2 V). (**d**) Nonlinearity factors and dynamic ranges obtained from the potentiation and depression characteristics at different V_G_. (**e**) Schematic diagram of the three-layer ANN model with each layer fully connected through the synaptic weights. (**f**) Hidden neuron-dependent recognition accuracy according to the different V_G_.

In addition, synaptic weight update characteristics are fundamental to memory and forgetting processes in the human system. The strengthening and weakening of the synaptic weight are represented by the potentiation and depression behaviors, respectively [52,53]. Figure 5a shows the updating of the synaptic weights in the optical potentiation and electrical depression (P/D) characteristics. The inset depicts the applied potentiation and depression stimuli conditions. The potentiation stimulus consists of 30 optical spikes (0.59 mW/cm^2^, 50 ms) under V_G_ of 0 V, and the depression stimulus involves 30 electrical spikes (–3 V, 50 ms) applied to the gate electrode. To read the change in the channel conductance, a read spike (1 V, 250 ms) was applied to the drain electrode. The P/D characteristics for optical spikes under different V_G_ values are shown in Figure S11 (Supplementary Materials). The channel conductance represents the synaptic weight, and it increases with the optical spikes and decreases with the electrical spikes. Figure 5b depicts the endurance of the P/D characteristics over five cycles. The cycle-to-cycle variation was <1%, confirming stability against repeated stimulation. In Figure 5c, the normalized P/D characteristics are obtained by dividing each channel conductance (G_n_) by the first one (G_1_) at V_G_ values of −0.2 V, 0 V, and 0.2 V. The normalized conductance values increase with the increase in V_G_, indicating that the synaptic weight update can be controlled through the EDL effect with V_G_ in our device. Meanwhile, high synaptic weight update linearity and a large dynamic range (DR) are important for achieving high recognition and learning accuracies [54,55]. The linearity property is described by a nonlinearity factor, which can be extracted as follows [56]:

(3) G=Gmin×GmaxGminw           ,         if α=0Gmaxα−Gminα×w+Gminα1/α,  if α≠0

where *w* is the internal variable (ranging from 0 to 1), and *G*_max_ and *G*_min_ represent the maximum and minimum conductance, respectively. α is the nonlinearity factor with an ideal value of 1: α_p_ for potentiation and α_d_ for depression. DR, which represents the range of synaptic weight updates, is calculated by dividing *G*_max_ by *G*_min_ [55]. Figure 5d shows the parameters obtained from the P/D characteristics at different V_G_ values. At a V_G_ of −0.2 V, α_p_, α_d_, and DR were 2.73, −0.68, and 1.93, respectively. Increasing V_G_ considerably improves both the linearity and DR. At a V_G_ of 0.2 V (α_p_, α_d_, and DR were 2.19, −0.41, and 2.99), α_p_ and α_d_ were closer to the ideal values and larger DR. To verify the learning and recognition ability of the neuromorphic systems in the suggested optoelectronic synaptic transistor, we constructed a three-layer ANN model using the calculated parameters and the normalized channel conductance, as shown in Figure 5e. The designed ANN model comprised three layers: the input, hidden, and output layers. The input and output layers contain 784 and 10 neurons, respectively, corresponding to an MNIST image with 28 × 28 pixels and ten digits (0–9). The hidden layer includes from 10 to 200 neurons. The ANN model was trained using 60,000 images from the handwritten MNIST training dataset, and the recognition accuracy was tested with 10,000 images. Figure 5f displays the accuracy as the recognition test results obtained at various values, varying the number of hidden neurons from 10 to 200. For 10 hidden neurons, the recognition accuracy for three cases, which were synaptic weight updates under a V_G_ of −0.2 V, 0 V, and 0.2 V, was 37.8%, 53.7%, and 56.4%, respectively. As the number of hidden neurons increased, recognition accuracy improved, and the best recognition accuracy was achieved with 200 hidden neurons. At 200 hidden neurons, the recognition accuracy was 87.9%, 90.3%, and 90.5% under a V_G_ of −0.2 V, 0 V, and 0.2 V, respectively. Therefore, the accuracy was influenced by the linearity and DR of the synaptic weight updates according to the V_G_. These results demonstrate a significant application potential of the IGZO optoelectronic synaptic transistor with a casein electrolyte-based EDL for visual neuromorphic computing.

## 4. Conclusions

In this work, we reported an IGZO optoelectronic synaptic transistor with a casein electrolyte-based EDL, which allows for the control of synaptic plasticity through the EDL effect. Significant synaptic properties were successfully emulated using the PPC effect in the IGZO channel upon illumination. The EDL effect of the casein electrolyte, induced by the migration of protons according to the V_G_, enabled the effective modulation of the recombination of photo-generated electrons and, consequently, the synaptic plasticity. Accordingly, EPSC, PPF, SRDP, and SNDP, representing synaptic plasticity, exhibited characteristic modulations with respect to V_G_. More importantly, the memory level was converted from STM to LTM by fine tuning the synaptic plasticity through the synergistic interplay of optical spikes and the EDL effect induced by V_G_. Furthermore, in recognition simulation utilizing the ANN model for the MNIST handwritten digits, we achieved a high recognition rate of 90.5%. These results show that the optoelectronic IGZO synaptic transistor with a casein electrolyte-based EDL has the potential to be applied to various technologies such as image recognition technology, wireless communication technology, and neuromorphic visual systems.

## Figures and Tables

**Table 1:** Performance comparison of the proposed IGZO optoelectronic transistor with oxide-based optoelectronic synaptic devices.

Reference	Active Layer	Device Type	Spike Wavelength	PPF Index	Energy Consumption
[25]	ZnO	Transistor	365 nm	~140% (NA)	~1 μJ
[44]	SnO_x_/HfO_x_	Memristor	405 nm	~138% (Δt = 5 s)	NA
[45]	In_2_O_3_/ZnO/FTO	Memristor	365 nm	~180% (Δt = 1 s)	~0.2 nJ
[46]	IAZO	Transistor	375 nm	~155.9% (Δt = 200 ms)	~2.3 pJ
[47]	PNCs/IGZO	Transistor	640 nm	~180% (Δt = 2 s)	~2.6 pJ
This works	IGZO	Transistor	395 nm	~168% (Δt = 200 ms)	~1.1 pJ

## Data Availability

Not applicable.

## Supplementary Materials

The following supporting information can be downloaded at https://www.mdpi.com/article/10.3390/biomimetics8070532/s1, Figure S1: (**a**) Optical image of IGZO optoelectronic synaptic transistor with a casein electrolyte-based EDL; Figure S2: (**a**) FTIR spectra of the casein electrolyte film ranging in wavenumber from 4000 cm^−1^ and 800 cm^−1^ [57,58,59]. (**b**) C-*f* characteristics of the casein electrolyte film using ITO/casein electrolyte-based EDL/ITO capacitor with frequency from 100 to 10^6^ Hz; Figure S3: Schematic of (**a**) biological synapse, and (**b**) EPSC evoked mechanism by the optical pulse in the proposed device; Figure S4: EPSC evoked by a single pre-synaptic optical spike (395 nm) with different light intensities of (**a**) 0.13 mW/cm^2^, (**b**) 0.31 mW/cm^2^, (**c**) 1.09 mW/cm^2^, and (**d**) 1.5 mW/cm^2^ for different spike widths (from 20 to 500 ms); Figure S5: EPSC triggered by a single pre-synaptic optical spike (395 nm, 0.59 mW/cm^2^) for different spike widths (from 20 to 500 ms) under (**a**) V_G_ of −0.2 V and (**b**) V_G_ of 0.2 V; Figure S6: EPSCs triggered by paired optical spikes (0.59 mW/cm^2^, 50 ms) with Δt of 200 ms under (**a**) V_G_ of −0.2 V and (**b**) V_G_ of 0.2 V; Figure S7: (**a**) EPSC showing minimum energy consumption for an optical spike (0.13 mW/cm^2^, 20 ms). (**b**) The energy consumption against the spike width; Figure S8: SRDP behavior triggered by sequential 10 pre-synaptic optical spikes (0.59 mW/cm^2^, 50 ms) with various frequencies from 0.1 to 8 Hz under (**a**) V_G_ of −0.2 V and (**b**) V_G_ of 0.2 V; Figure S9: SNDP behaviors with various numbers of optical spikes (0.59 mW/cm^2^, 50 ms) under (**a**) V_G_ of −0.2 V and (**b**) V_G_ of 0.2 V; Figure S10: Synaptic weight decay characteristic fitted with a stretched exponential function (Equation (2)); Figure S11: P/D characteristics for optical potentiation spike (0.59 mW/cm^2^, 50 ms) under (**a**) V_G_ of −0.2 V and (**b**) V_G_ of 0.2 V and electrical depression spike (−3 V, 50 ms).
