# Supplementary material for: Light-Stimulated IGZO Transistors with Tunable Synaptic Plasticity Based on Casein Electrolyte Electric Double Layer for Neuromorphic Systems
Source: Biomimetics (Basel). 2023 Nov 9;8(7):532. doi: 10.3390/biomimetics8070532 (PMC10669183; doi:10.3390/biomimetics8070532)
Supplement: Supplementary file 1 [file biomimetics-08-00532-s001.zip › biomimetics-2610336-Supplementary Materials.pdf]

Supplementary Materials

Light-Stimulated IGZO Transistors with Tunable Synaptic Plasticity Based on Casein Electrolyte Electric Double Layer for Neuromorphic Systems

Hwi-Su Kim [1], Hamin Park [2] and Won-Ju Cho [1,*]

[1] Department of Electronic Materials Engineering, Kwangwoon University, Gwangun-ro 20, Nowon-gu, Seoul 01897, Republic of Korea; hwisu0811@naver.com
[2] Department of Electronic Engineering, Kwangwoon University, Gwangun-ro 20, Nowon-gu, Seoul 01897, Republic of Korea; parkhamin@kw.ac.kr
* Correspondence: chowj@kw.ac.kr; Tel.: +82-2-940-5163

(a)

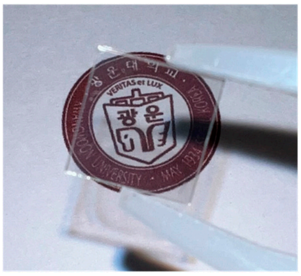

Figure S1. (a) Optical image of IGZO optoelectronic synaptic transistor with a casein electrolyte-based EDL.

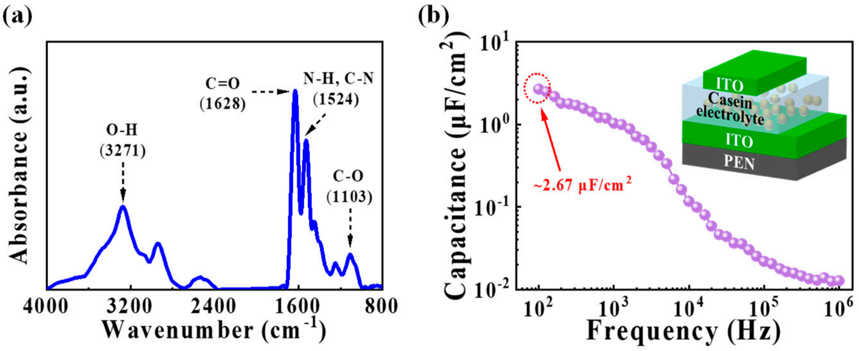

Figure S2. (a) FTIR spectra of the casein electrolyte film ranging in wavenumber from 4000 cm^{-1} and 800 cm^{-1}. (b) C-*f* characteristics of the casein electrolyte film using ITO/casein electrolyte-based EDL/ITO capacitor with frequency from 100 Hz to 10^6 Hz.

Figure S2a shows the analyzed chemical compositions of the casein electrolyte film. The broad peak at 3271 cm^{-1} is attributed to the O-H stretching. The peak near 1628 cm^{-1} is related to the C=O stretching. The peak at 1524 cm^{-1} is due to the N-H bending and C-N stretching, respectively. The peak at 1103 cm^{-1} is caused by the C-O stretching [1–3].

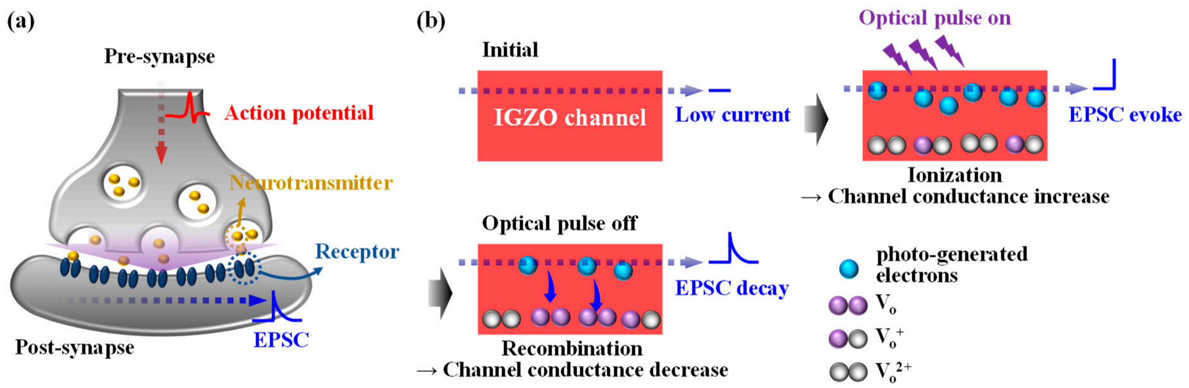

Figure S3. Schematic of (a) biological synapse, and (b) EPSC evoked mechanism by the optical pulse in the proposed device.

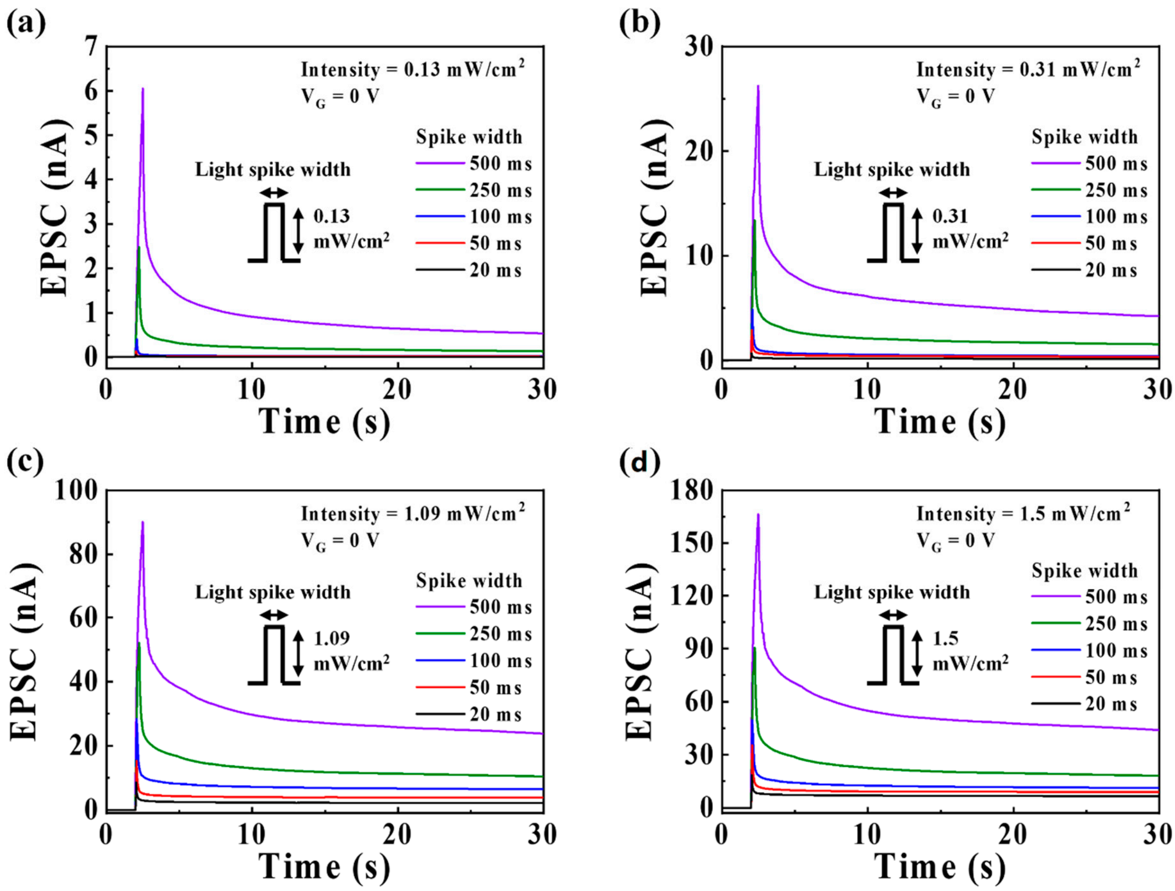

Figure S4. EPSC evoked by a single pre-synaptic optical spike (395 nm) with different light intensities of (a) 0.13 mW/cm^2, (b) 0.31 mW/cm^2, (c) 1.09 mW/cm^2, and (d) 1.5 mW/cm^2 for different spike widths (from 20 ms to 500 ms).

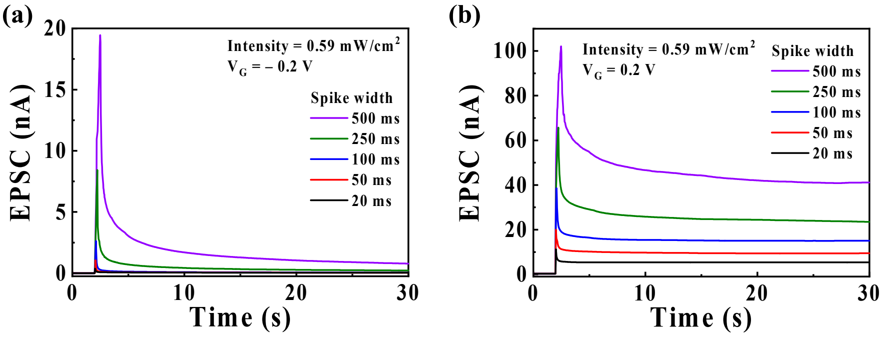

Figure S5. EPSC triggered by a single pre-synaptic optical spike (395 nm, 0.59 mW/cm^2) for different spike widths (from 20 ms to 500 ms) under (a) V_G of -0.2 V and (b) V_G of 0.2 V.

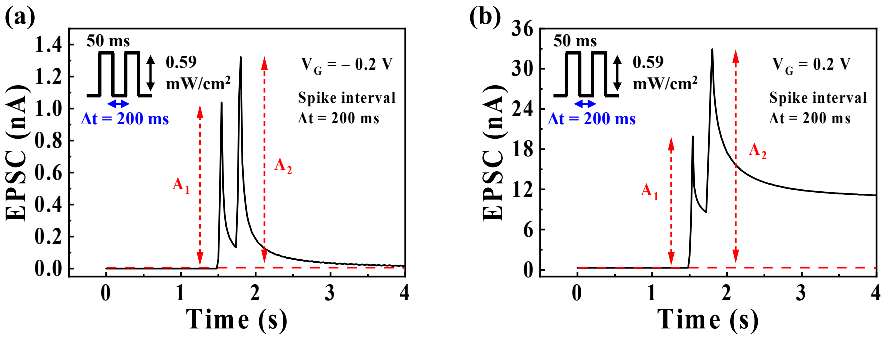

Figure S6. EPSCs triggered by paired optical spikes (0.59 mW/cm^2, 50 ms) with Δt of 200 ms under (a) V_G of -0.2 V and (b) V_G of 0.2 V.

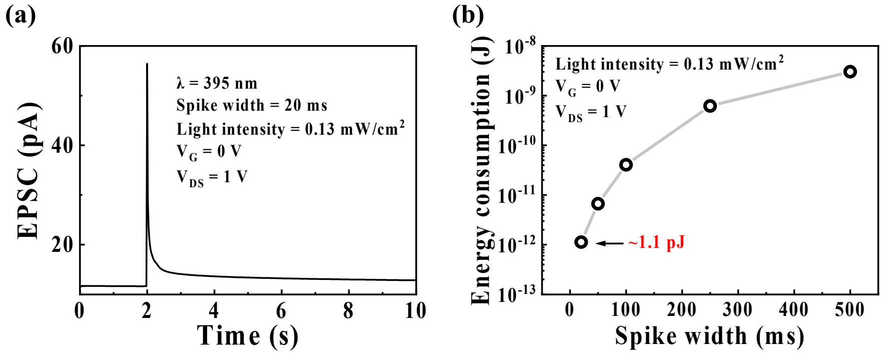

Figure S7. (a) EPSC showing minimum energy consumption for an optical spike (0.13 mW/cm^2, 20 ms). (b) The energy consumption against the spike width.

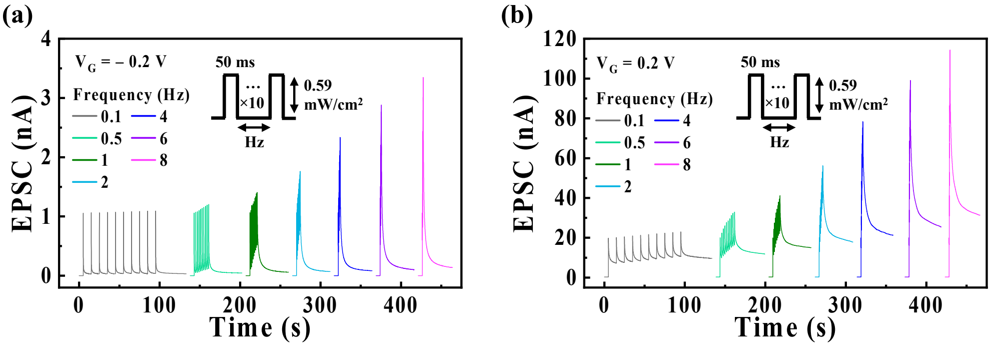

Figure S8. SRDP behavior triggered by sequential 10 pre-synaptic optical spikes (0.59 mW/cm^2, 50 ms) with various frequencies from 0.1 Hz to 8 Hz under (a) V_G of -0.2 V and (b) V_G of 0.2 V.

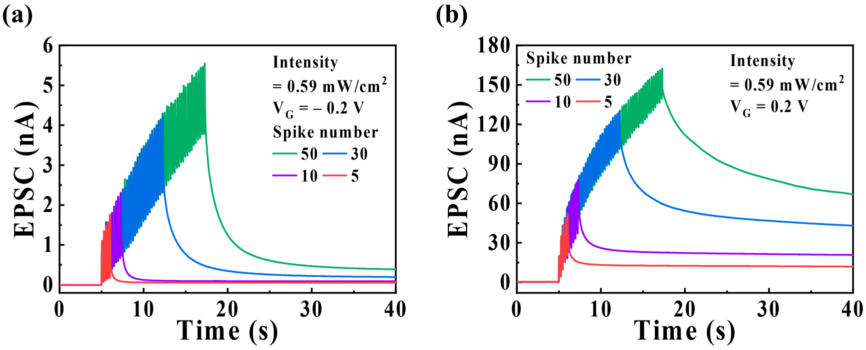

Figure S9. SNDP behaviors with various numbers of optical spikes (0.59 mW/cm^2, 50 ms) under (a) V_G of -0.2 V and (b) V_G of 0.2 V.

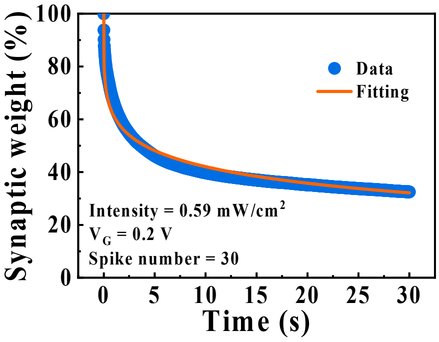

Figure S10. Synaptic weight decay characteristic fitted with a stretched exponential function (Eq. 2).

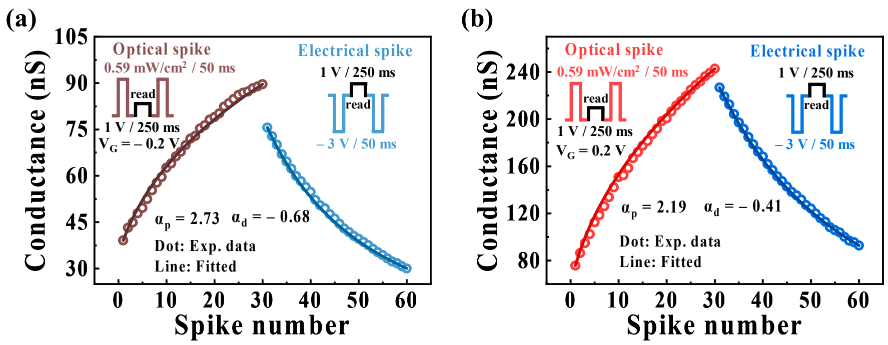

Figure S11. P/D characteristics for optical potentiation spike (0.59 mW/cm^2, 50 ms) under (a) V_G of -0.2 V and (b) V_G of 0.2 V and electrical depression spike (-3 V, 50 ms).

References

[1] Curley, D.M.; Kumosinski, T.F.; Unruh, J.J. and Farrell Jr, H.M. Changes in the secondary structure of bovine casein by Fourier transform infrared spectroscopy: Effects of calcium and temperature, J. Dairy Sci. 1998, 81(12), pp.3154-3162.

[2] Shao, F.; Cai, M.L.; Gu, X.F. and Wu, G.D. Starch as ion-based gate dielectric for oxide thin film transistors, Org. Electron. 2017, 45, pp.203-208.

[3] Szyk-Warszyńska, L.; Raszka, K. and Warszyński, P. Interactions of casein and polypeptides in multilayer films studied by FTIR and molecular dynamics, Polymers 2019, 11(5), p.920.
